# Comparison of early versus late rescue stenting after failed thrombectomy for intracranial atherosclerosis-related large vessel occlusion

**DOI:** 10.1007/s00701-025-06470-2

**Published:** 2025-02-25

**Authors:** In-Hyoung Lee, Sung-Kon Ha, Dong-Jun Lim, Jong-Il Choi

**Affiliations:** Department of Neurosurgery, Korea University Ansan Hospital, Korea University College of Medicine, 123 Jeokgeum-Ro, Danwon-Gu, Ansan, Gyeonggi-Do 15355 South Korea

**Keywords:** Acute ischemic stroke, Recanalization, Stents, Thrombectomy, Intracranial atherosclerosis

## Abstract

**Background:**

The optimal timing for rescue stenting (RS) following failed thrombectomy in patients with underlying intracranial atherosclerosis (ICAS)-related large vessel occlusion (LVO) remains unknown. We aimed to determine this by comparing the angiographic and clinical outcomes of early and late RS.

**Methods:**

We retrospectively enrolled patients with acute ischemic stroke who underwent stent-retriever thrombectomy for ICAS-related LVO. Per-pass analysis was performed to assess recanalization rates after each retrieval in all patients. Patients were classified into early and late groups based on the number of stent retrievals before RS (early: 1 or 2 attempts; late: ≥ 3 attempts). Angiographic and clinical outcomes were compared between these two groups.

**Results:**

Among 126 patients with ICAS-related LVO, 257 retrievals were evaluated. Successful and complete recanalization rates were highest on the first attempt and significantly decreased between the second and third passes. Overall, 56 patients underwent RS, of which 27 and 29 were classified into the early and late RS groups, respectively. The early RS group had shorter procedure times (45 min vs. 70 min, *p* < 0.001) and higher rates of favorable outcomes (85.2% vs. 55.2%, *p* = 0.014) than the late RS group. Intact stent patency rates were also higher in the early RS group than in the late RS group (88.0% vs. 65.4%, *p* = 0.059). Multivariate analysis identified early RS (OR, 7.187; 95% CI, 1.385–37.306; *p* = 0.019) and stent patency (OR, 7.291; 95% CI, 1.288–41.277; *p* = 0.025) as significant factors influencing favorable outcomes.

**Conclusion:**

RS should be performed at an early stage after failed thrombectomy for ICAS-related LVO.

## Introduction

Stent retriever thrombectomy (SRT) is currently considered a mainstay treatment strategy in patients with acute ischemic stroke (AIS) caused by anterior circulation large-vessel occlusion (LVO) since several randomized clinical trials (RCTs) have demonstrated that timely SRT is a safe and clinically beneficial treatment [5, 7, 13]. However, the previous RCTs have not adequately addressed the outcomes in patients with underlying intracranial atherosclerosis (ICAS), one of the leading causes of LVO. Patients with LVO attributed to underlying ICAS generally tend to have SRT resistance, leading to substantially reduced reperfusion rates and increased re-occlusion frequencies; therefore, it is common for them to encounter therapeutic challenges in real-world clinical practice [3, 8, 15]. Despite this, the optimal therapeutic strategy for ICAS-related LVO remains unclear. Recent studies have demonstrated the safety and feasibility of the rescue stenting (RS) technique as a rescue procedure for patients with ICAS-related LVO after thrombectomy failure [3, 4, 32]. However, it is currently unclear how many retrieval attempts should be performed before deciding to terminate repeated thrombectomy and utilizing the RS technique in cases of refractory occlusion. Therefore, we aimed to determine the optimal timing of RS after a failed thrombectomy for ICAS-related LVO by comparing the angiographic and clinical outcomes of two different stages of performing RS (early *vs*. late). For the present study, we hypothesized that (1) the rate of recanalization success after each stent retrieval will decrease with an increasing number of attempts, and (2) repeated stent retrieval prior to RS may be ineffective in refractory cases in patients diagnosed with ICAS-related LVO.

## Methods and materials

### Patient enrollment and data acquisition

We retrospectively identified consecutive patients with AIS owing to intracranial LVO in the anterior circulation who were treated by SRT at our tertiary institution between January 2017 and December 2023, according to the current national guidelines [25]. Our institution’s ethical committee approved the current study. Written informed consent was waived because of its retrospective nature.

**Fig. 1:**
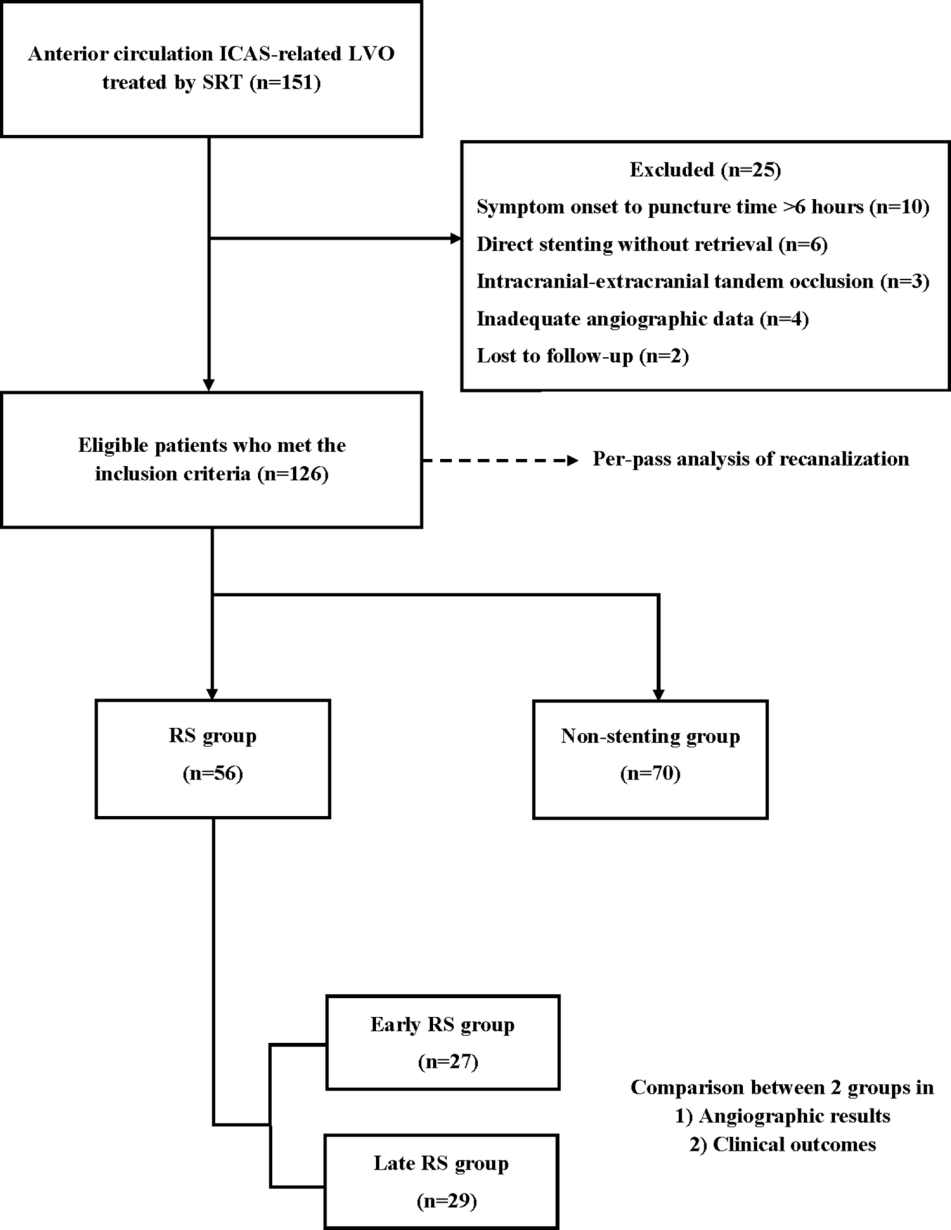
Flowchart of the patient selection process. ICAS, intracranial atherosclerosis; LVO, large vessel occlusion; SRT, stent-retriever thrombectomy; RS, rescue stenting

For the current study, we only extracted data from patients deemed ICAS-related LVO, defined by the following angiographic findings discovered during thrombectomy: (1) truncal-type occlusion, (2) fixed stenosis exceeding 50% on post-deployment angiography, and (3) remnant stenosis or re-occlusion tendency after recanalization [2, 4, 23]. In addition, we focused this investigation on the occlusion of the proximal middle cerebral artery (MCA-M1) and intracranial internal carotid artery (ICA). We excluded patients who (1) underwent thrombectomy 6 h after the symptom onset, (2) underwent direct stenting without retrieval of a stent, (3) were diagnosed with intracranial-extracranial tandem occlusion, (4) were lacking adequate angiographic data to determine the presence of ICAS, and (5) were lost to follow-up. The ICAS-related LVO patients were initially dichotomized into two groups: SRT without RS or with RS. After performing a per-pass analysis of recanalization in overall ICAS-related LVO patients, the patients who were treated using the RS technique were classified into two groups depending on the number of stent retrievals performed before RS. The early RS group was defined as permanent stent placement after either a single or two passes of stent-retriever. The late RS group was defined as permanent stenting after three or more passes of stent-retriever. Figure 1 shows the flowchart for the current study’s patient selection process.

The following data were retrieved and analyzed: demographics, medical history, clinical characteristics (baseline National Institutes of Health Stroke Scale score, Alberta Stroke Program Early CT Score, occlusion site of vessel, and time from stroke onset to groin puncture), and procedure details (procedure time, intravenous thrombolysis, and number of stent retrieval attempts).

### Intervention procedure

Stroke-trained neurologists performed intravenous thrombolysis on eligible patients before the procedure in accordance with the national guidelines [25]. All procedures were performed by three experienced, dual-trained neurovascular surgeons using a biplane neuro-angiography suite with conscious sedation. The use of a balloon guide catheter (mainly Cello, Medtronic) was preferred; however, this was determined according to the preference of the attending physician. Alternatively, a 6-Fr Shuttle sheath (Cook Medical) was chosen as the guiding catheter. The use of intermediate catheter (SOFIA, Microvention) for concurrent aspiration was at the discretion of attending neurovascular surgeons. The occluded vessel was navigated and passed using a 0.021-inch microcatheter (Phenom 21, Medtronic) with the assistance of a 0.014-inch microwire. Selective angiography was then performed using a microcatheter to define the occlusion site, following which a thrombectomy procedure was performed using stent retriever devices. The type of stent mainly used was a Solitaire FR (Medtronic), which is capable of retrieval and detachment and can be used as both a front-line device for SRT and a permanent stent if necessary. The total number of retrieval attempts was not restricted and was left to the operator’s discretion. RS was considered when successful recanalization was not achieved or residual stenosis persisted after the serial retrieval process. RS was performed as follows: after a stent was temporarily deployed for approximately 15 min, serial angiography was conducted to assess antegrade flow and adequate coverage of the target stenotic lesion. During this RS procedure, adjuvant intra-arterial glycoprotein IIb/IIIa inhibitor (tirofiban) was infused through a microcatheter. The total dosage of tirofiban typically ranged from 0.5 mg to 1.0 mg [17]. Immediately before stent detachment, intact stent patency and improvement in residual stenosis were confirmed by delayed angiography. Balloon angioplasty before or after permanent stenting was not performed in our institutions. Intravenous tirofiban was only administered continuously for 12 h after the procedure in cases where hemorrhagic complications were ruled out on the brain CT scan performed immediately after the procedure. Subsequently, dual antithrombic medications (100 mg aspirin and 75 mg clopidogrel) were provided orally or through a nasogastric tube to all patients in whom hemorrhagic complications were ruled out on follow-up brain imaging study.

### Angiographic and clinical outcomes

Angiographic results were assessed based on the modified Thrombolysis in Cerebral Infarction (mTICI) score in a postprocedural recanalization state. A final cerebral angiography demonstrating a mTICI score of 2b or 3 was considered successful recanalization.[30] Stent patency and distal blood flow of the stented vessel were evaluated using follow-up CT angiography or MR angiography, which was obtained before discharge and 3 months after the endovascular treatment. The stented vessel was deemed as “patent” when it had an intact distal circulation without re-occlusion. Clinical outcomes included functional outcomes, mortality, and the occurrence of symptomatic intracranial hemorrhage (ICH). Functional outcomes were assessed by stroke-trained neurologists using the modified Rankin Scale (mRS) scores at 3 months post-treatment. We defined mRS scores ≤ 2 as a favorable functional outcome. ICH detected on postprocedural brain CT scans performed both immediately after and within 3 days after the procedure was regarded as symptomatic if the NIHSS score increased by 4 points or more from baseline [20].

### Statistical analysis

We compared the likelihood of per-pass successful and complete recanalization according to the number of stent retrievals in overall ICAS-related LVO patients. Patients who underwent more than 4 stent retrieval attempts were combined into one category. The patients treated with the RS technique were categorized into two groups according to the number of stent retrieval attempts prior to RS, and their baseline clinical characteristics, angiographic results, and clinical outcomes were evaluated. Student’s t-test or Mann–Whitney U test was used to analyze continuous variables. The results were presented as mean with standard deviation or median with interquartile range. Categorical variables, expressed as frequencies, were analyzed using Pearson’s chi-square statistic or Fisher’s exact test, as appropriate. Multivariate logistic regression analysis was performed to assess each variable’s contribution to favorable outcomes. Variables with a *p* value of < 0.1 in the univariate analysis were entered in the multivariate analysis as candidate variables. A *p* value of < 0.05 was considered statistically significant. All statistical analyses were conducted using the SPSS 25.0 software (IBM, Armonk).

## Results

### Per pass analysis of recanalization

**Fig. 2:**
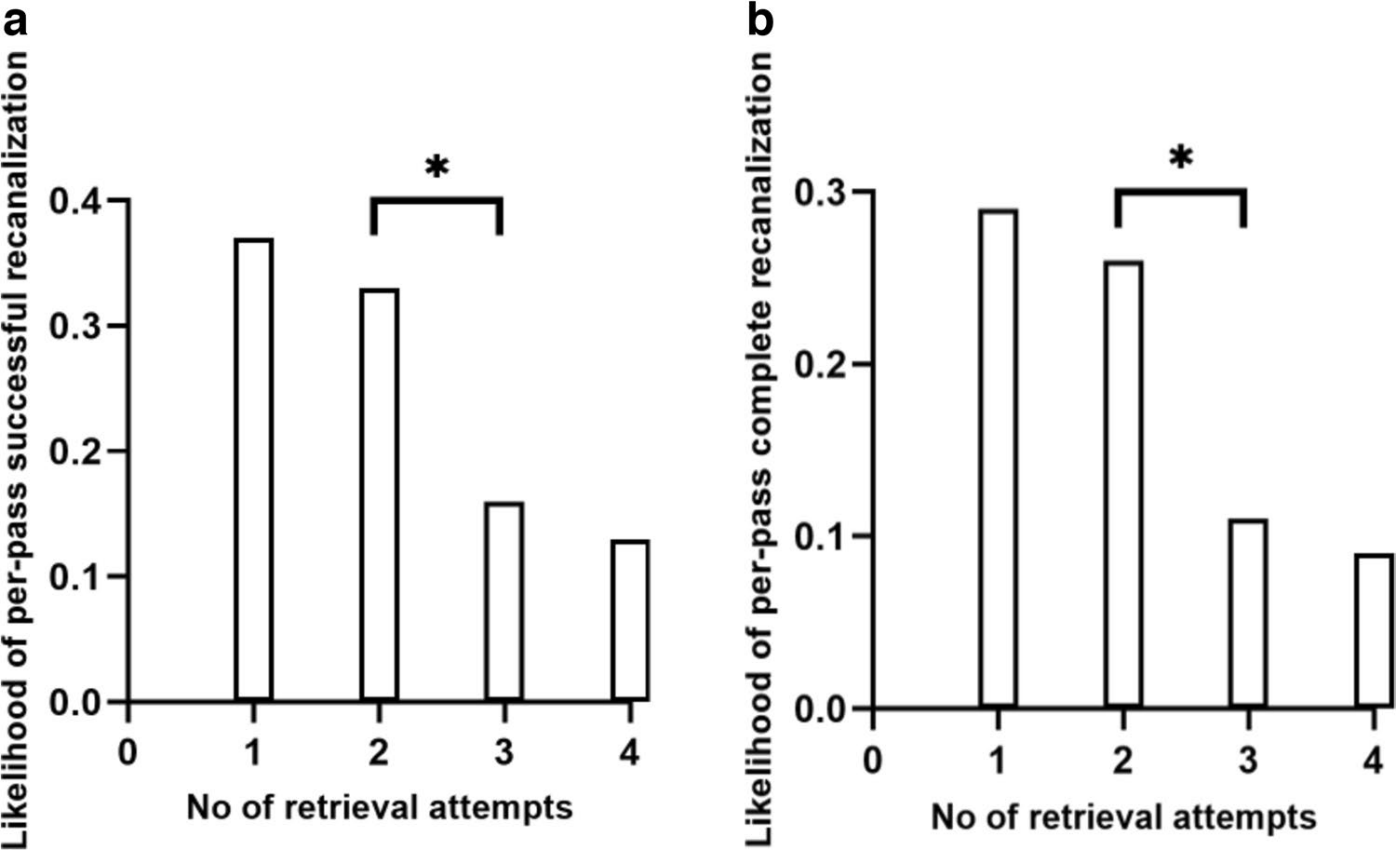
Association of the number of stent retrievals with the per-pass likelihood of (**A**) successful recanalization (mTICI 2b or 3) and (**B**) complete recanalization (mTICI 3). mTICI, modified Thrombolysis in Cerebral Infarction

The current study enrolled 126 consecutive AIS patients with ICAS-related LVO who met the inclusion criteria. A total of 257 passes were evaluated. Figure 2A shows the per-pass likelihood of achieving successful recanalization (mTICI 2b or 3), which was highest for the first pass (47/126, 37.3%) and gradually declined after the first pass. Between the second and the third passes of a stent, there was a significant decrease in the likelihood of successful recanalization (32.9% *vs*. 15.7%, *p* = 0.040). The per-pass analysis for complete recanalization showed similar results (Fig. 2B). The likelihood of obtaining complete recanalization was highest at the first pass (36/126, 28.6%) and decreased for the second and subsequent passes. Notably, the difference between the second and the third passes was significant (25.7% *vs*. 10.5%, *p* = 0.040).

### Comparisons of demographics and treatment outcomes between the early and late RS groups

During the study period, 56 patients underwent RS for thrombectomy failure. Of these, 27 patients received RS after either a single or two passes of stent-retriever (early RS group), whereas 29 patients underwent permanent stenting after more than three passes (late RS group). Table 1 provides a summary of the baseline and clinical characteristics of both groups. There were no significant intergroup differences, except for the total attempts of stent retrieval.

**Table 1:** Demographics and clinical characteristics – differences between the early and late RS groups

	Early RS (*n* = 27)	Late RS (*n* = 29)	*p*-value
Sex, male	16 (59.3)	18 (62.1)	0.834
Age, years, mean (SD)	70.4 ± 11.3	68.8 ± 12.7	0.624
Medical history			
Hypertension	14 (51.9)	14 (48.3)	0.794
Diabetes mellitus	7 (25.9)	6 (20.7)	0.651
Dyslipidemia	4 (14.8)	4 (13.8)	0.915
Current smoking	9 (33.3)	9 (31.0)	0.857
Previous ischemic stroke	4 (14.8)	3 (10.3)	0.623
Atrial fibrillation	2 (7.4)	2 (6.9)	0.942
Antiplatelet medication	6 (22.2)	7 (24.1)	0.868
Baseline NIHSS score, median (IQR)	10 (8–14)	11 (9–15)	0.275
ASPECTS, median (IQR)	8 (8–9)	9 (8–9)	0.460
Site of occlusion			0.639
Intracranial ICA	5 (18.5)	4 (13.8)	
MCA—M1	22 (81.5)	25 (86.2)	
Onset-to-puncture, min, mean (SD)	264.8 ± 52.6	254.7 ± 52.1	0.471
Intravenous thrombolysis	7 (25.9)	10 (34.5)	0.494
Use of balloon guiding catheter	13 (48.1)	17 (58.6)	0.442
Use of intermediate catheter	7 (25.9)	6 (20.7)	0.651
Total number of stent retrieval attempts, mean (SD)	1.4 ± 0.5	3.7 ± 0.8	< 0.001*
1	16 (59.3)		
2	11 (40.7)		
3		14 (48.3)	
≥ 4		15 (51.7)	

Data are presented as numbers (percentages) unless otherwise indicated. *Statistically significant

*RS;* Rescue stenting, *SD;* Standard deviation, *NIHSS;* National Institutes of Health Stroke Scale, *IQR;* Interquartile range, *ASPECTS;* Alberta Stroke Program Early CT Score, *ICA;* Internal carotid artery, *MCA* Middle cerebral artery

The disparities in angiographic and clinical outcomes between the two groups are summarized in Table 2. The procedure time was significantly shorter in the early RS group than in the late RS group (45 min vs. 70 min, *p* < 0.001). While the final recanalization result and rate of successful recanalization were comparable in both groups regardless of the timing of performing RS, there was a tendency toward a higher rate of intact stent patency on follow-up with the early RS group than in the late RS group (88.0% vs. 65.4%, *p* = 0.059). Furthermore, the early RS group achieved a significantly higher rate of favorable outcomes than the late RS group (85.2% vs. 55.2%, *p* = 0.014). The rate of symptomatic ICH in the early RS group was lower compared with the late RS group, but this difference did not reach statistical significance (3.7% vs. 17.2%, *p* = 0.105).

**Table 2:** Angiographic and clinical outcomes – differences between the early and late RS groups

	Early RS (*n* = 27)	Late RS (*n* = 29)	*p*-value
Angiographic outcomes			
Procedure time, min, median (IQR)	45 (40–55)	70 (55–80)	< 0.001*
Final mTICI			0.465
1	0 (0)	0 (0)	
2a	2 (7.4)	4 (13.8)	
2b	12 (44.4)	13 (44.8)	
3	13 (48.1)	12 (41.4)	
Successful recanalization	25 (92.6)	25 (86.2)	0.445
Intact stent patency on follow-up**	22/25 (88.0)	17/26 (65.4)	0.059
Clinical outcomes			
Favorable outcome	23 (85.2)	16 (55.2)	0.014*
Mortality	2 (7.4)	3 (10.3)	0.705
Symptomatic ICH	1 (3.7)	5 (17.2)	0.105

Data are presented as numbers (percentages) unless otherwise indicated. *Statistically significant. **Stent patency follow-up was available for 25 patients in the early RS group and 26 in the late RS group

*RS;* Rescue stenting, *SD;* Standard deviation, *IQR;* Interquartile range, *mTICI;* Modified thrombolysis in cerebral infarction, *ICH* Intracranial hemorrhage

### Factors influencing favorable outcomes after RS

Univariate analysis revealed that age (*p* = 0.045), baseline NIHSS score (*p* = 0.070), successful recanalization (*p* = 0.041), intact stent patency on follow-up (*p* = 0.094), and early RS (*p* = 0.014) were associated with favorable outcomes; therefore, these factors were entered into the multivariate analysis. In the multivariate analysis, intact stent patency on follow-up (OR, 7.291; 95% CI, 1.288–41.277; *p* = 0.025) and early RS (OR, 7.187; 95% CI, 1.385–37.306; *p* = 0.019) were significantly influencing an increased likelihood of favorable functional outcomes (Table 3).

**Table 3:** Factors influencing favorable functional outcomes in patients who underwent RS

	Favorable outcome (*n* = 39)	Unfavorable outcome (*n* = 17)	*p*-value (univariate)	Odds ratio (95% CI)	*p*-value (multivariate)
Sex, male	26 (66.7)	8 (47.1)	0.191		
Age, years, mean (SD)	67.6 ± 12.3	74.1 ± 10.0	0.045	0.951 (0.884–1.022)	0.171
Medical history					
Hypertension	20 (51.3)	8 (47.1)	0.779		
Diabetes mellitus	7 (17.9)	6 (35.3)	0.163		
Dyslipidemia	6 (15.4)	2 (11.8)	0.718		
Current smoking	12 (30.8)	6 (35.3)	0.751		
Previous ischemic stroke	4 (10.3)	3 (17.6)	0.497		
Atrial fibrillation	2 (5.1)	2 (11.8)	0.384		
Baseline NIHSS score, median (IQR)	10 (8–12.5)	14 (10–16)	0.070	0.923 (0.774–1.100)	0.368
ASPECTS, median (IQR)	9 (8–9)	8 (8–9)	0.859		
Site of occlusion			0.841		
Intracranial ICA	6 (15.4)	3 (17.6)			
MCA—M1	33 (84.6)	14 (82.4)			
Onset-to-puncture, min, mean (SD)	253.3 ± 54.7	260.3 ± 39.9	0.597		
Intravenous thrombolysis	13 (33.3)	4 (23.5)	0.459		
Use of balloon guiding catheter	22 (56.4)	8 (47.1)	0.534		
Use of intermediate catheter	9 (23.1)	4 (23.5)	0.972		
Successful recanalization	37 (94.9)	13 (76.5)	0.041	2.549 (0.463–4.044)	0.282
Intact stent patency on follow-up	32 (82.1)	7 (41.2)	0.094	7.291 (1.288–41.277)	0.025*
Early RS (vs. late RS)	23 (59.0)	4 (23.5)	0.014	7.187 (1.385–37.306)	0.019*

Data are presented as numbers (percentages) unless otherwise indicated. *Statistically significant

*RS;* Rescue stenting, *SD;* Standard deviation, *NIHSS;* National Institutes of Health Stroke Scale, *IQR;* Interquartile range, *ASPECTS*; Alberta Stroke Program Early CT Score, *ICA;* Internal carotid artery, *MCA;* Middle cerebral artery

## Discussion

To the best of our knowledge, the present study is the first investigation to evaluate the per-pass rate of recanalization targeting only ICAS-related LVO and to explore the optimal timing of RS in failed thrombectomy by comparing the treatment outcomes of two different stages of performing RS. The present study demonstrated two main findings: (1) the likelihood of angiographic recanalization significantly decreased after the second stent retrieval in patients with ICAS-related LVO, and (2) early RS, performed after a single or two attempts of stent retrieval, offered significant advantages over late RS in terms of procedural efficacy and clinical outcomes.

Several recent studies have focused on the threshold number of thrombectomy maneuvers that correlate with better angiographic and clinical outcomes. A preliminary study based on this insight reported that successful recanalization after the third attempt did not result in favorable clinical outcomes [10]. Similarly, a recent meta-analysis indicated that the likelihood of successful recanalization dropped after the third pass of the device [1]. The other authors advocated that the probability of angiographic improvement and achieving favorable outcomes significantly decreases after the second thrombectomy attempt [18, 27]. Although controversy remains regarding the specific number of maneuvers at which the risk of successive thrombectomy outweighs the benefits, there is consensus on the detrimental effects of multiple thrombectomy attempts on angiographic results and clinical outcomes, which is consistent with our results [1, 11, 12, 18].

In patients with LVO due to underlying ICAS, rescue treatment is often required because of re-occlusion tendency and residual stenosis following an initial recanalization of the occluded artery [15, 19, 23]. Re-occlusion tendency may originated from the pathophysiology of ICAS-related LVO, most likely due to in-situ thrombo-occlusion caused by rupture of unroofed atherosclerotic plaque [23]. Several techniques, including the use of aspiration devices, permanent intracranial stenting, balloon angioplasty, and intra-arterial thrombolysis, have been considered as rescue treatments for lesions refractory to conventional thrombectomy procedures [6, 28]. Among these, RS, inspired by studies that explored the efficacy and risks of permanent stenting in patients with ICAS [9], is an emerging option that is valuable when encountering refractory cases, and recent studies have shown its safety and significant benefits in clinical outcomes in patients with failed thrombectomy [4, 16, 24]. In regarding the rate of successful recanalization, a recent multicenter registry reported that 98.7% of patients were successfully recanalized by performing RS [4]. In consistent with our results, which reported a successful recanalization rate of 89.3%, another study also showed that about 87% of patients experienced successful recanalization through RS after initial thrombectomy failure [24]. However, it is an overlooked issue how many thrombectomy attempts should be made in cases where the first pass of the device failed before aborting the thrombectomy procedure and performing RS. This decision is mainly made at the operator’s discretion and is not based on statistical evidence. Consequently, the results of our analysis, revealing that early RS is advantageous over late RS in terms of recanalization and clinical outcomes, may serve as an indicator for future research on the optimal treatment strategy for refractory ICAS-related LVO.

The potential mechanisms supporting the superiority of RS performed at an early stage for failed thrombectomy may be explained by various clinical scenarios. First, multiple stent retrievals after re-occlusion tend to be less effective and have a higher probability of periprocedural complications because they may cause vessel injury at the stenotic site and could result in vasospasm, dissection, and refractory occlusion [19, 23, 29]. Additionally, repeated manipulations of a microwire and microcatheter may provoke accumulated vascular endothelial injury and atherosclerotic plaque fragmentation, leading to re-occlusion [4, 17]. Another concern of repeated stent retrieval is the risk of hemorrhagic complications due to vessel rupture or tearing of the perforating vessel [14, 29]. In line with previous studies reporting that multiple passes with a stent retriever increase the risk of postprocedural hemorrhage, our results showed a tendency for a higher rate of symptomatic ICH in patients who received RS at a later stage [14, 31]. Another possible explanation for worsened clinical outcomes in the late RS group may be the delayed recanalization related to additional retrieval before RS. In the current analysis, the procedure time was significantly shorter in the early RS group than in the late RS group, which was an expected result based on the number of stent retrieval attempts before permanent placement. This is in line with previous research indicating that a longer procedure time is correlated with poor outcomes after thrombectomy [21, 26].

Recent studies have reported the safety and efficacy of direct stent placement as a “first-line” rather than a “rescue” treatment strategy after failed thrombectomy for ICAS-related LVO [17, 29]. They propose that direct stenting without thrombectomy may decrease the procedure time and reduce procedure-related complications such as vessel injury, vasospasm, or postprocedural hemorrhage. However, given the possibility that permanent stenting may not be necessary in successfully recanalized cases without re-occlusion with single or two attempts of stent retrieval, direct stenting is rarely performed in our institutions. Moreover, there are several inherent concerns regarding permanent stent placement. First, permanent stenting requires aggressive antiplatelet treatment to prevent in-stent thrombosis and preserve its patency. These antiplatelet medications may potentially increase the rate of hemorrhagic complications despite previous studies having reported a comparable rate of SICH between the stenting group and the non-stenting group [3, 22]. Second, stent-related ischemic complications could arise after permanent stenting. For example, the thrombus might be compressed by the stent, resulting in thrombus fragments that could obstruct the adjacent perforator [33]. Therefore, attempting stent retrieval in a single or two passes for ICAS-related LVO is a reasonable treatment strategy for successful recanalization without permanent stent placement; however, if thrombectomy fails after two retrievals, we suggest aborting the repeated retrieval and performing RS. We propose that future research focusing on the comparisons between stenting as a rescue treatment and direct stent placement in the treatment of ICAS-related LVO should be performed.

Despite its various strengths, the present study had some limitations. The relatively small, retrospectively extracted data may limit the generalizability of the findings. Additionally, selection bias may have been introduced because the timing of RS was not randomized and the decision of when to perform RS depended on the operator’s discretion. Moreover, although the safety and efficacy of balloon angioplasty have been reported [22, 32], it was not performed at our institution, which is a methodological limitation. Another significant issue is the determination of ICAS-related LVO. Unfortunately, there are still no uniform criteria for diagnosing underlying ICAS. Moreover, it might be challenging to discriminate underlying ICAS from remnant thrombi following thrombectomy on cerebral angiography. While our study’s angiographic definition is reliable because we included sustained fixed stenosis on post-deployment angiography, further investigation is required to refine a more precise and standard definition of underlying ICAS in patients with LVO. Finally, our results cannot be generalized in the patients who underwent thrombectomy in an extended time window because the patients whose interval between symptom onset and puncture exceeded 6 h were excluded from this study. Despite these limitations, the current study offers an intriguing insight into the optimal number of retrievals prior to RS in failed thrombectomy. However, future randomized studies are warranted to validate our results and establish standardized protocols for the optimal timing of performing RS in patients with ICAS-related LVO.

## Conclusions

The current study revealed that early RS performed after a single or two failed stent retrieval attempts offers significant advantages over late RS regarding angiographic and clinical outcomes in patients with ICAS-related LVO. Therefore, we suggest that RS should be performed at an early stage after failed SRT in these patients.

## Data Availability

No datasets were generated or analysed during the current study.
